# A Simple Method for Purification of Vestibular Hair Cells and Non-Sensory Cells, and Application for Proteomic Analysis

**DOI:** 10.1371/journal.pone.0066026

**Published:** 2013-06-04

**Authors:** Meike Herget, Mirko Scheibinger, Zhaohua Guo, Taha A. Jan, Christopher M. Adams, Alan G. Cheng, Stefan Heller

**Affiliations:** 1 Department of Otolaryngology – HNS, Stanford University, Stanford, California, United States of America; 2 Department of Molecular and Cellular Physiology, Stanford University, Stanford, California, United States of America; 3 Mass Spectrometry Core, Stanford University, Stanford, California, United States of America; Universitat Pompeu Fabra, Spain

## Abstract

Mechanosensitive hair cells and supporting cells comprise the sensory epithelia of the inner ear. The paucity of both cell types has hampered molecular and cell biological studies, which often require large quantities of purified cells. Here, we report a strategy allowing the enrichment of relatively pure populations of vestibular hair cells and non-sensory cells including supporting cells. We utilized specific uptake of fluorescent styryl dyes for labeling of hair cells. Enzymatic isolation and flow cytometry was used to generate pure populations of sensory hair cells and non-sensory cells. We applied mass spectrometry to perform a qualitative high-resolution analysis of the proteomic makeup of both the hair cell and non-sensory cell populations. Our conservative analysis identified more than 600 proteins with a false discovery rate of <3% at the protein level and <1% at the peptide level. Analysis of proteins exclusively detected in either population revealed 64 proteins that were specific to hair cells and 103 proteins that were only detectable in non-sensory cells. Statistical analyses extended these groups by 53 proteins that are strongly upregulated in hair cells versus non-sensory cells and vice versa by 68 proteins. Our results demonstrate that enzymatic dissociation of styryl dye-labeled sensory hair cells and non-sensory cells is a valid method to generate pure enough cell populations for flow cytometry and subsequent molecular analyses.

## Introduction

Molecular analyses of the inner ear’s specialized cell types are hindered by the paucity of these cells. This fact might be one of the reasons why hearing and balance are among the senses that are still only partially elucidated at the molecular level. Although a single inner ear contains several thousand sensory hair cells, the cells are scattered into five vestibular sensory patches plus a sixth auditory sensory epithelium located in the cochlea. This spatial dispersion combined with the circumstance that the inner ear is shielded by one of the hardest bones of the body makes it difficult to obtain sufficient quantities of sensory hair cells and their associated supporting cells for molecular analysis. Obviously, sensory hair cells are interesting because present-day research seeks to understand the process of mechanoelectrical transduction, or pursues the specific proteins that contribute to the unique features of the hair cells’ afferent ribbon synapses, among a battery of other interesting topics surrounding hair cell biology [1], [2]. Supporting cells, on the other hand, are interesting because in non-mammalian vertebrates they appear to serve as somatic stem cells, able to reverse vestibular and cochlear hair cell loss and restore function [3]. In mammals, only a few supporting cells of the adult vestibular sensory epithelia display stem cell characteristics [4], whereas cochlear supporting cells lose this feature during the first neonatal weeks [5]–[7].

Creative use of transgenic mice in combination with flow cytometry is a recently utilized strategy for purification of hair cells [7], supporting cells [6], [8], [9], and other otic cell types [10], [11] for molecular and other cell biological analyses. Likewise, fluorescently labeled antibodies to cell surface proteins have also been used for purification of various cell populations from the inner ear [7], [12]. Despite many advantages of these two strategies, they have the disadvantage of requiring either a transgenic reporter or the expression of a specific cell surface marker on the cell type of interest. We sought to develop a strategy that eliminates these requirements by utilizing a functional feature of mature sensory hair cells - their ability to rapidly take up certain styryl dyes [13], [14]. In addition, we used the avian inner ear utricle and saccule, two vestibular organs whose sensory maculae can be enzymatically detached and peeled away from underlying cells, allowing the harvest of sensory epithelia that consist solely of hair cells, and non-sensory cells including supporting cells. We chose to analyse the purified cell populations by mass spectrometry, which unveiled a snapshot of the proteomic profiles of vestibular hair cells and non-sensory cells. We utilized a statistical data analysis strategy that was valuable in dealing with potential cross-contamination, which we identified as a potential limitation of the technology. Our overall strategy led to the identification of more than one hundred proteins each specific for hair cells and non-sensory cells demonstrating the applicability of styryl dye labeling and flow cytometry for inner ear research.

## Results and Discussion

### Dissociation of vestibular sensory epithelia into single cells

**Figure 1:**
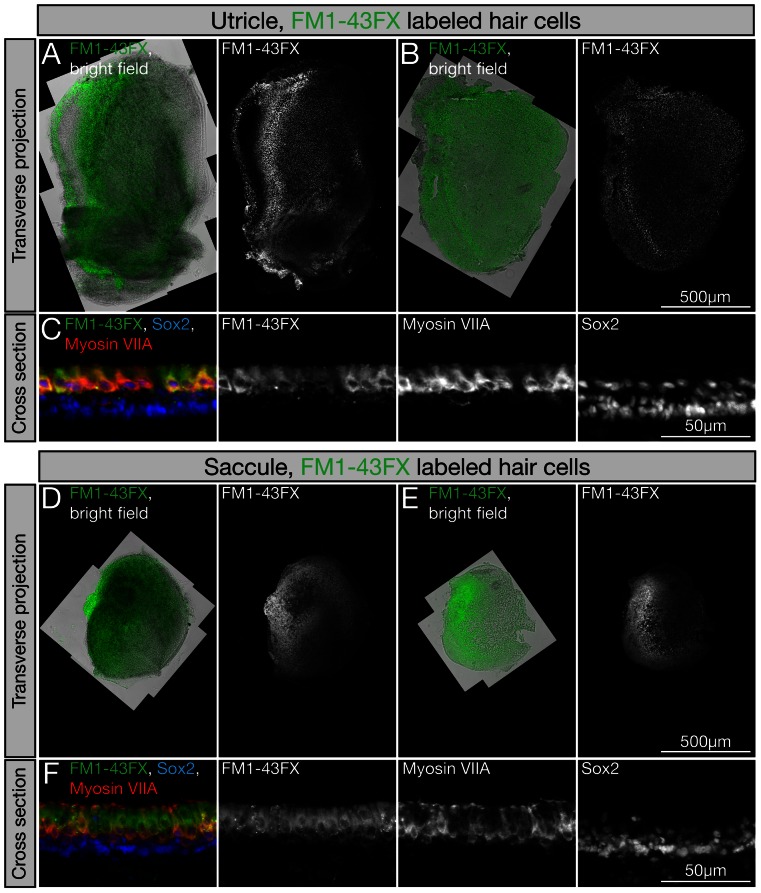
Uptake of styryl dye by vestibular hair cells. (A,B,D,E) Styryl dyes AM1-43 and FM1-43FX distinctively label sensory hair cells of utricles and saccules (images for FX1-43FX are shown). (B,E) After incubation in thermolysin, stained sensory epithelia of utricles and saccules were peeled off the underlying stromal tissue. Shown are transverse projections of utricle whole mounts and peeled sensory epithelia of utricles and saccules. To better visualize styryl dye labeled tissue, we combined light microscopic images and fluorescence FM1-43FX visualization. (C,F) Shown are a cross sections of E18 chicken utricle and saccule with FM1-43FX labeled hair cells (green), co-labeled with antibodies to the hair cell marker myosin VIIa (red) and Sox2 (blue), which is detectable in hair cells and supporting cells.

We used chicken embryos at their 18^th^ day of incubation for isolation of hair cells, non-sensory and supporting cells. We focused on the vestibular maculae of the utricle and saccule for three reasons: i) they comprise the largest hair cell-bearing organs of the inner ear containing more than 20,000 hair cells per utricular macula, ii) the hair cells are functional at this late embryonic age [15], and iii) utricles and saccules can be dissected relatively quickly in larger numbers. After dissection and removal of otolithic membranes, the tissues were exposed to the styryl dye AM1-43 or FM1-43FX (Fig. 1A,D). Brief exposure to either of these dyes intensely labels living hair cells, whereas supporting and other non-sensory cells remain unlabeled [13] (Fig. 1C,F). Differentially labeling of hair cells and non-sensory cells is the basis for subsequent separation of hair cells from residual unlabeled cells of the sensory epithelia by flow cytometry. Specificity of the dye-uptake was confirmed by immunocytochemistry with antibodies to the hair cell marker myosin VIIA (Fig. 1C, F). After hair cell labeling, the sensory epithelia of the utricles and saccules were enzymatically detached from underlying stromal cells, mechanically separated from the stromal layer, and the living epithelia consisting of labeled hair cells and unlabeled non-sensory cells including supporting cells were collected in fresh media (Fig. 1B, E).

**Figure 2:**
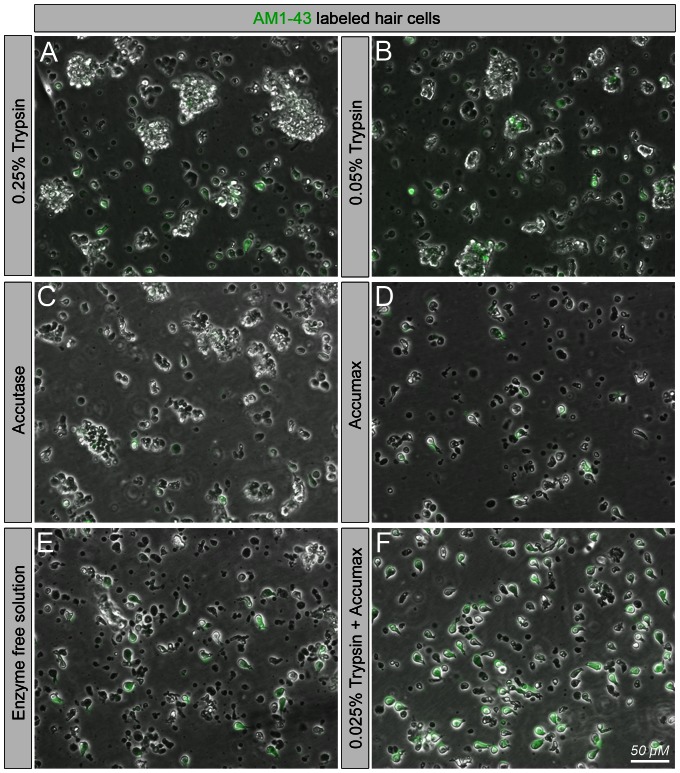
Dissociation of vestibular sensory epithelia into single hair cells and non-sensory cells. AM1-43-stained sensory epithelia underwent different enzymatic and non-enzymatic treatments to test for optimal conditions to separate hair cells and non-sensory cells and to preserve hair cell morphology. Conditions were: 0.25% trypsin (A), 0.05% trypsin (B), accutase (C), accumax (D), enzyme-free (E) and 50% accumax + 0.025% trypsin (F). Shown are representative images of cells after mild trituration following 7 min incubations at 37°C.

We optimized the dissociation method for vestibular sensory epithelia to ensure thorough cell separation and minimal cell aggregation but also high viability judged by cell shape and exclusion of propidium iodide, a dye that is generally unable to enter viable cells. Representative results obtained with different dissociation strategies are shown in Figure 2. The predominantly enzymatic digestion conditions included 0.25% trypsin, Accutase cell detachment mixture, an enzyme-free formulation of chelating reagents, 0.05% trypsin, Accumax cell dissociation mixture, and 50% Accumax plus 0.025% trypsin. We found that neither trypsin alone nor the commercially available enzyme cocktails Accutase or Accumax were satisfactory to quantitatively dissociate the tissue. These tests were systematically done by varying incubation times from a few minutes to up-to 30 minutes, followed by mild trituration, and resulted in either insufficient cell separation or starkly reduced viability (Fig. 2A–D). Combining Accumax cell dissociation solution at half strength with a low concentration of trypsin, however, for a total incubation time of 7 minutes resulted in the optimal separation of viable individual cells (Fig. 2F). Hair cells separated with this procedure displayed at least some rudimentary preservation of cytomorphology. The enzyme-free formulation of chelating reagents alone was also highly efficient for cell separation (Fig. 2E); however, hair cell morphology was not well preserved in this condition, presumably caused by chelating divalent cations such as Ca^2+^, which are important for hair bundle integrity [16], [17]. Moreover, cell viability was reduced when compared with the Accumax and trypsin combination.

### Flow cytometric purification of AM1-43 labeled hair cells and unlabeled non-sensory cells

**Figure 3:**
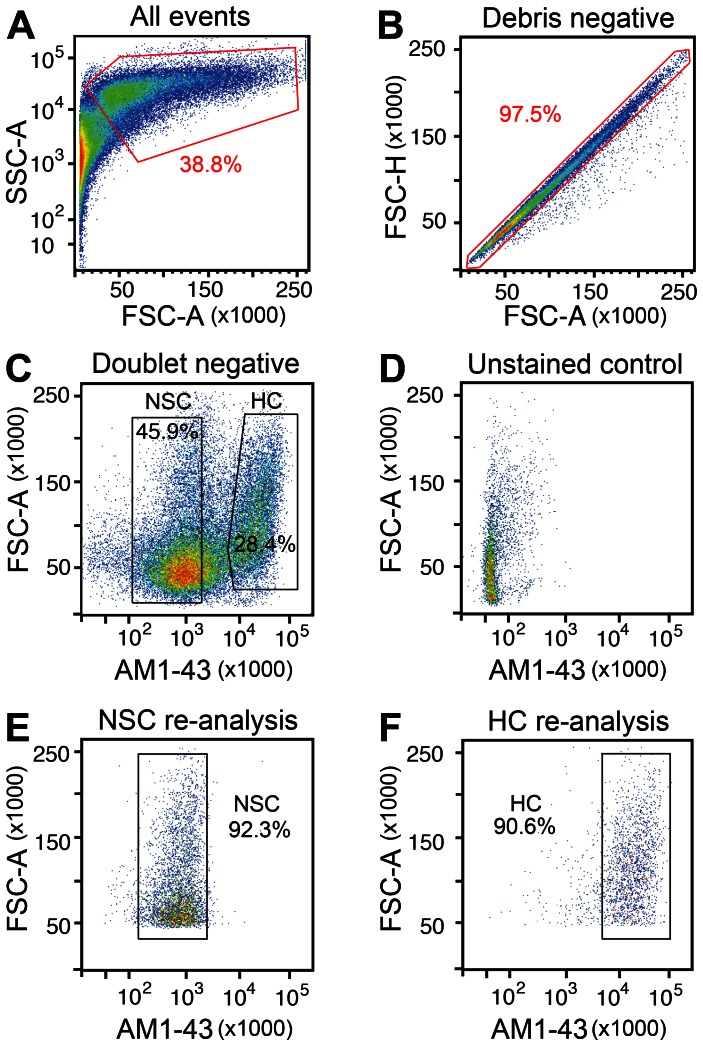
Flow cytometric separation of AM1-43 labeled and unlabeled cells. Single cell suspensions generated from AM1-43-exposed vestibular sensory epithelia were subjected to one-color fluorescence-activated cell sorting. (A) to (C) depicts the gating strategy: (A) cell debris was excluded on based low side (SSC) and forward scatter (FSC) parameters. (B) From the debris-negative population, doublets were removed based on their divergence from a linear FSC-height and FSC-area gate. (C) From the debris-free and doublet-negative population, AM1-43-high cells (HC, presumptive hair cells) and AM1-43-low cells (NSC, non-sensory cells) were gated for collection. (D) Analysis of unstained cells revealed a single population and no AM1-43 fluorescence background. (E,F) Re-sort analyses of the two populations shown in (C), demonstrated >90% purity.

After cell dissociation, intense AM1-43 labeling of hair cells persisted (Fig. 2F), which we utilized to separate the AM1-43-positive cells from unstained cells. The flow cytometric gating strategy disregarded propidium iodide-labeled dead cells, which ranged between 7–15%, as well as cell debris (Fig. 3A). Doublets were identified and excluded based on non-proportionate forward scatter for height and area parameters (Fig. 3B). Of the viable single cells, AM1-43-high and AM1-43-low cell populations were gated for collection (Fig. 3C). We expected that the AM1-43-high population consist of labeled hair cells, whereas the AM1-43-low population should comprise mainly supporting cells, contaminating mesenchymal cells from the underlying stroma, undifferentiated/progenitor cells, and perhaps some immature or damaged hair cells that did not take up the styryl dye. These potential contaminants are not a limitation of the embryonic age of the tissue because undifferentiated and immature cells are also detectable in posthatch chickens [15]. Approximately 20–25% cells displayed AM1-43 fluorescence intensities between the low and high gates and were not collected. To demonstrate specificity, we used vestibular sensory epithelia not exposed to AM1-43 dye as a negative control and subjected the dissociated cells to flow cytometric analysis. With this control, we only detected a single population of viable single cells that displayed background levels of fluorescence in the AM1-43 detection channel (Fig. 3D). In 6 experiments using approximately 120±30 utricles and saccules per independent experiment, we collected on average 31.4±8.8% AM1-43-high, presumed hair cells, and on average 43.3±9.5% AM1-43-low, presumed non-sensory cells. In numbers, this corresponds to 172,200±60,000 hair cells and 261,700±100,400 non-sensory cells per experiment. When re-sorted with the same parameters, each of the populations displayed at least 90% purity (Fig. 3E,F).

### Mass spectrometry and proteomic analyses revealed distinct proteomic signatures of hair cells and non-sensory cells

The AM1-43-high and AM1-43-low populations of each sorting experiment were collected into lysis buffer, concentrated and the proteins of each population were electrophoretically separated. Eight polyacrylamide gel pieces representing eight electrophoretically fractionated groups of proteins were excised for each cell population, in-gel digested with trypsin, and analyzed by liquid chromatography-tandem mass spectrometry (LC-MS/MS) for protein identification and quantification. In total, we obtained three independent datasets for AM1-43-high and AM1-43-low cell populations, respectively.

**Figure 4:**
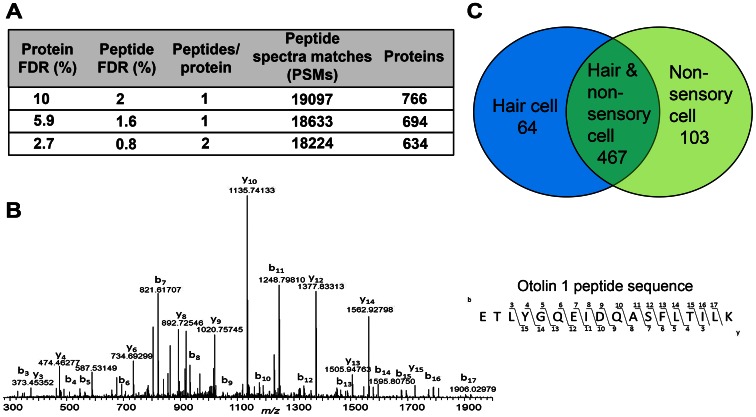
Shotgun proteomic analysis of isolated hair cells and non-sensory cells. (A) False discovery rates (FDR) at both protein and peptide levels were applied to filter the data to a representative number of PSMs, which result in the number of proteins identified. The majority of proteins were represented by more than one PSM. (B) Demonstration of a MSMS peptide spectral match (PSM) of the peptide ETLYGQEIDQASFLTILK from the protein otolin-1 (OTOL1). The peptide was identified with better than 2 parts-per-million (ppm) mass accuracy, where the experimental mass/charge (*mz)* was 1035.0452 Da and the theoretical 1035.0437 Da. The annotated b and y ions are indicative of peptide backbone bond cleavage between carbonyl carbon and nitrogen, whereas in the case of the amino-terminus, b-ions result, and for the carboxyl-terminus, y-ions. OTOL1 was only identified in the AM1-43-low cell fraction (see Fig. 3C), and suggests that this protein is present in non-sensory cells, presumably supporting cells, and not in hair cells. (C) Venn diagram displaying the number of proteins unique to hair cells (64), non-sensory cells (104) and the number of proteins shared between the two cell types (467) at the stringent filter setting of requiring at least 2 different peptides per protein. This corresponds to the FDR settings shown in the third line of the table shown in (A).

For the interpretation of the resulting mass spectrometry based datasets, refined statistical search strategies that mitigate and control incorrect peptide identifications were used. Specifically, we utilized concatenated target-decoy database searches, which employ a strategy using composite protein target sequence databases and decoy sequences that comprise the reversed target sequences to estimate false positive identification rates for individual peptide-spectral matches (PSMs) [18]. With this method, it is possible to distinguish between correctly identified spectra, which should be derived solely or mainly from target sequences, and incorrectly identified PSMs, which should be traceable more or less in equal proportions to target and decoy sequences. Based on the resulting hits, a false discovery rate (FDR) was generated for each PSM dataset, which we used to filter matching PSMs (Fig. 4A).

We further used spectral counting as a quantitative tool to assemble expression profiles of proteins detectable in hair cell and supporting cell fractions. Spectral counting relies on the identification of peptide spectra at the tandem mass spectrometry fragment ion level and sums the number of spectra identified for a given protein (Fig. 4B). The results for all detected peptides are integrated and reported as a single count value for a particular protein. One of the major drawbacks in relying on spectral counting data is that its quantitative power at a low number of counts can be unreliable. To address this issue to the best of our ability, and in dealing with data derived from very low cell counts, a minimum of two peptides identified for a particular protein was set as a prerequisite. When we applied these criteria, we were able to identify and provide semi-quantitative abundance values for 634 proteins. These hits were based on 18,224 PSMs with a protein FDR of 2.7% and a peptide FDR of 0.8%, respectively (Fig. 4A). We acknowledge that more exact quantitative measurements would be needed for more precise data analysis, for example summed dissociation-product ion-current intensities [19] or isobaric tags [20] combined with sophisticated normalization and standardization [21].

### tringent analyses revealed proteins that are specifically detectable in hair cells and non-sensory cells

The majority (467) of the 634 identified proteins were detected in AM1-43-high as well as in AM1-43-low cell populations (Fig. 4C). 64 proteins were only detected in AM1-43-high cells and 103 proteins were found only in AM1-43-low cells (Fig. 4C and Tables 1 and 2). Among the proteins specifically detected in the AM1-43-high cell population (presumed hair cells), seven were identified in three of three experiments. These proteins were myosin VIIa (MYO7A), glutathione-S-transferase homolog (GSTO1), proteasome subunit alpha (PSMA1), toll-like receptor 3 (TLR3), acyl-CoA hydrolase (ACOT7), star-related lipid transfer domain 10 (STARD10) and the secretory carrier membrane protein 1 (SCAMP1). Myosin VIIA is a commonly used hair cell marker [22], [23], which confirms that the AM1-43-high cell population indeed contained hair cells.

**Table 1:** Proteins exclusively identified in hair cells.

Hair Cell Only Identified Proteins	Accession Number	Experiments Observed (total of 3)	Sum Spectral Count	Hair Cell Only Identified Proteins	Accession Number	Experiments Observed (total of 3)	Sum Spectral Count
MYO7A similar to Myosin VIIA	IPI00576099	3	21	SNRPA1 U2 small nuclear ribonucleoprotein A	IPI00575703	2	3
GSTO1 similar to glutathione-S-transferase homolog isoform 2	IPI00593631	3	15	TOLLIP Toll-interacting protein	IPI00590435	2	3
PSMA1 Proteasome subunit alpha type-1	IPI00820937	3	8	LOC770724 NADH dehydrogenase [ubiquinone] 1 beta subcomplex subunit 6	IPI00602158	2	3
TLR3 Toll-like receptor 3	IPI00590386	3	7	HIP1 Huntingtin-interacting protein 1	IPI00818913	1	5
ACOT7 similar to acyl-CoA hydrolase	IPI00571165	3	6	SLC17A8 similar to vesicular glutamate transporter 3	IPI00579531	1	4
STARD10 StAR-related lipid transfer (START) domain	IPI00579939	3	6	20 kDa protein Mesencephalic astrocyte-derived neurotrophic factor precursor	IPI00602683	1	4
SCAMP1 similar to secretory carrier membrane protein 1	IPI00589108	3	6	ARL1 ADP-ribosylation factor-like 1	IPI00578232	1	3
CRABP1 Cellular retinoic acid-binding protein 1	IPI00602403	2	13	NDUFS4 similar to NADH dehydrogenase	IPI00597417	1	3
PGM2L1 Phosphoglucomutase 2-like 1	IPI00594777	2	13	TARDBP TAR DNA-binding protein 43	IPI00596633	1	3
PSMB1 Proteasome subunit beta type-1	IPI00583929	2	11	64 kDa protein Synapsin-3	IPI00578493	1	3
RAB7A similar to RAB7 protein	IPI00601244	2	10	TMEM35 Transmembrane protein 35	IPI00571302	1	3
RCJMB04_3m23 Vesicle-associated membrane protein-associated protein A	IPI00819526	2	9	APBA1 similar to adaptor protein X11alpha	IPI00580720	1	3
RAB2A Ras-related protein Rab-2A	IPI00582079	2	7	RCJMB04_1g4 Serine/arginine-rich splicing factor 10 isoform 2	IPI00584494	1	3
OSBPL1A similar to oxysterol-binding protein-like 1A isoform 2	IPI00582014	2	7	HSPH1 Heat shock protein 105 kDa	IPI00590633	1	3
PSMB2 Proteasome subunit beta type-2	IPI00588689	2	7	USP7 Ubiquitin specific peptidase 7	IPI00580665	1	3
RCJMB04_32c11 Elongation factor 1-beta	IPI00597497	2	7	ITPA Inosine triphosphate pyrophosphatase	IPI00594943	1	2
LOC776238 similar to rabconnectin	IPI00599229	2	6	MYL1 Myosin light chain 1, skeletal muscle isoform	IPI00578052	1	2
Eukaryotic translation initiation factor 5A-1	IPI00577746	2	6	KIF21A Kinesin family member 21A	IPI00588407	1	2
NDUFV2 similar to NADH dehydrogenase [ubiquinone] flavoprotein 2	IPI00571196	2	6	APOA1BP Apolipoprotein A-I binding protein	IPI00576049	1	2
LPGAT1 Lysophosphatidylglycerol acyltransferase 1	IPI00587613	2	6	ATP5I ATP synthase, H+ transporting	IPI00576667	1	2
NDUFS3 NADH dehydrogenase [ubiquinone] Fe-S protein 3 precursor	IPI00572839	2	6	RBBP4 Histone-binding protein RBBP4	IPI00592914	1	2
SNRPB Small nuclear ribonucleoprotein-associated protein B'	IPI00603436	2	5	ATP6V1H similar to 54 kDa vacuolar H(+)-ATPase subunit	IPI00593252	1	2
COX7A2L similar to cytochrome c oxidase polypeptide VIIa-heart	IPI00579138	2	5	ACSL4 similar to Acyl-CoA synthetase	IPI00593747	1	2
ATP5H ATP synthase subunit d	IPI00594088	2	4	BRWD2 Bromodomain and WD repeat-containing protein 2	IPI00594946	1	2
COX4I1 Cytochrome c oxidase subunit IV	IPI00576496	2	4	YKT6 Synaptobrevin homolog YKT6	IPI00597412	1	2
NDUFB4 NADH dehydrogenase [ubiquinone] 1 beta subcomplex subunit 4	IPI00812364	2	4	AIFM1 Apoptosis-inducing factor 1, mitochondrial	IPI00601063	1	2
SLC1A6 similar to neuronal glutamate transporter EAAT4	IPI00594618	2	4	RPL9 60S ribosomal protein L9	IPI00601775	1	2
LRP8 Low-density lipoprotein receptor-related protein 8	IPI00581287	2	4	STARD8 StAR-related lipid transfer (START) domain containing 8	IPI00812461	1	2
ABHD10 Abhydrolase domain containing 10	IPI00602566	2	4	ACP1 Low molecular weight phosphotyrosine protein phosphatase	IPI00578195	1	2
RPL24 similar to Ribosomal protein L24	IPI00586190	2	3	USO1 General vesicular transport factor p115	IPI00578084	1	2
ATP1B1 Sodium/potassium-transporting ATPase subunit beta-1	IPI00579860	2	3	INPP5F Phosphatidylinositide phosphatase SAC2	IPI00577046	1	
EFCAB6 EF-hand calcium binding domain 6	IPI00596390	2	3				

Listed are proteins that were exclusively identified in hair cells as well as the number of times they were observed in three independent experiments and the sum of their spectral counts.

**Table 2:** Proteins exclusively identified in non-sensory cells.

Non-Sensory Cells Only Identified Proteins	Accession Number	Experiments Observed (total of 3)	Sum Spectral Count	Non-Sensory Cells Only Identified Proteins	Accession Number	Experiments Observed (total of 3)	Sum Spectral Count
CCT5 T-complex protein 1 subunit epsilon (TCP1)	IPI00575509	3	14	TTC38 Tetratricopeptide repeat protein 38	IPI00589671*	1	4
ANXA6 Annexin A6	IPI00576535	3	14	PRKAR1A cAMP-dependent protein kinase type I-alpha regulatory subunit	IPI00573783 *	1	4
LOC429161 similar to otolin-1	IPI00591329	3	12	CRMP1 Collapsin response mediator protein-1A	IPI00579627 *	1	4
PPP2R1B Protein phosphatase 2, regulatory subunit A, beta	IPI00811766	3	8	MOSC2 similar to MOCO sulphurase C-terminal domain containing 2	IPI00591218*	1	4
ALAD Delta-aminolevulinic acid dehydratase	IPI00600895	3	5	GNAI2 Guanine nucleotide-binding protein G(i) subunit alpha-2	IPI00589157*	1	4
LOC395261 Filamin	IPI00591901	2	21	RCJMB04_1g23 Cytoplasmic dynein 1 light intermediate chain 2	IPI00585015	1	4
DDOST Dolichyl-diphosphooligosaccharide-protein glycosyltransferase	IPI00602654	2	19	RCJMB04_7k22 Septin9	IPI00592494	1	4
CKAP4 Cytoskeleton-associated protein 4	IPI00584755	2	13	NCSTN Nicastrin	IPI00572509*	1	3
PSMD13 26S proteasome non-ATPase regulatory subunit 13	IPI00601716	2	12	PDHA1 Pyruvate dehydrogenase E1	IPI00595745*	1	3
IMMT Mitochondrial inner membrane protein	IPI00595381	2	7	RCJMB04_1d17 Regulation of nuclear pre-mRNA domain-containing protein 1B	IPI00651204*	1	3
ACAD9 Acyl-CoA dehydrogenase family member 9	IPI00821733	2	6	ANP32A Acidic leucine-rich nuclear phosphoprotein 32 family member A	IPI00589812*	1	3
SDHA Succinate dehydrogenase	IPI00682371	2	6	ADD1 Alpha-adducin	IPI00602199*	1	3
EIF3E Eukaryotic translation initiation factor 3 subunit E	IPI00593255	2	6	MAN2B2 similar to mannosidase, alpha, class 2B, member 2	IPI00572503*	1	3
RCJMB04_9j22 RNA binding motif protein, X-linked	IPI00575141	2	6	GNA11 Guanine nucleotide-binding protein G11 alpha-subunit	IPI00577333*	1	3
SMC1 Structural maintenance of chromosomes protein 1A	IPI00601137	2	5	EIF2S1 Eukaryotic translation initiation factor 2 subunit 1	IPI00590033*	1	3
USP5 Ubiquitin carboxyl-terminal hydrolase 5	IPI00579016	2	5	POFUT1 GDP-fucose protein O-fucosyltransferase 1	IPI00592268*	1	3
LRPAP1 Low density lipoprotein receptor-related protein associated protein 1	IPI00588285	2	5	UGP2 UTP—glucose-1-phosphate uridylyltransferase	IPI00601449*	1	3
13 kDa protein Desru_0254	IPI00818044	2	5	EEA1 Early endosome antigen 1	IPI00571138*	1	3
SEPT2 Septin-2	IPI00584652	2	5	BZW2 Basic leucine zipper and W2 domain-containing protein 2	IPI00577749 *	1	3
ATP2A2 Sarcoplasmic/endoplasmic reticulum calcium ATPase 2 (SERCA2)	IPI00590859	2	4	ATP1B3 Sodium/potassium-transporting ATPase subunit beta-3	IPI00580874*	1	3
ARHGDIB similar to D4-GDP-dissociation inhibitor	IPI00588997	2	4	STAG2 similar to stromal antigen 2	IPI00599733 *	1	3
RRBP1 Ribosome-binding protein 1	IPI00573911	2	4	RCJMB04_12m17 Short/branched chain specific acyl-CoA dehydrogenase	IPI00602866*	1	3
CSE1L similar to cellular apoptosis susceptibility protein	IPI00582808	2	4	PSMC1 26S protease regulatory subunit 4	IPI00821206*	1	3
TMC6 Transmembrane channel-like protein 6	IPI00679585	2	3	PITPNB Phosphatidylinositol transfer protein, beta	IPI00581857 *	1	3
96 kDa protein Solute carrier family 12 member 2 (NKCC1)	IPI00600831	2	3	HHATL Hedgehog acyltransferase-like	IPI00599649	1	3
CKM Creatine kinase M-type	IPI00592568	2	3	RCJMB04_1m9 Thyroid hormone receptor associated protein 3	IPI00583448*	1	2
AKT1 Serine/threonine protein kinase	IPI00582661	2	3	ATP13A3 similar to type V P-type ATPase	IPI00593562	1	2
TF Ovotransferrin	IPI00683271*	1	17	COL18A1 collagen, type XVIII, alpha 1	IPI00596507*	1	2
HADH similar to L-3-hydroxyacyl-Coenzyme A dehydrogenase	IPI00682714	1	11	PCYOX1 Prenylcysteine oxidase 1 precursor	IPI00573599*	1	2
PSMD6 26S proteasome non-ATPase regulatory subunit 6	IPI00601017	1	11	TALDO1 Transaldolase	IPI00571239*	1	2
LOC395260 Chicken gizzard actin-binding protein260	IPI00593882*	1	10	PA2G4 similar to proliferation-associated protein 1, partial	IPI00597630*	1	2
792 kDa protein Nesprin 2	IPI00585154	1	9	FDPS Farnesyl pyrophosphate synthase	IPI00584175*	1	2
COL8A2 Collagen, type VIII, alpha 2	IPI00584704*	1	8	PSMD11 26S proteasome subunit p44.5	IPI00598610*	1	2
PRPS1 Ribose-phosphate pyrophosphokinase 1	IPI00599017*	1	8	TXNDC10 Protein disulfide-isomerase TMX3	IPI00574033*	1	2
SEC61A1 similar to sec61-like protein	IPI00594100*	1	7	C14orf149 Proline racemase-like	IPI00574864*	1	2
SERPINB14B Ovalbumin-related protein Y	IPI00573738*	1	7	ERGIC1 Endoplasmic reticulum-golgi intermediate compartment (ERGIC) 1	IPI00575314*	1	2
ACTR3 Actin-related protein 3	IPI00587398*	1	7	COPS4 COP9 signalosome complex subunit 4	IPI00578250*	1	2
P4HA1 similar to Prolyl 4-hydroxylase alpha-1 subunit	IPI00598417*	1	7	SCLY Selenocysteine lyase	IPI00585168*	1	2
CSNK2A1 Casein kinase II subunit alpha	IPI00584282*	1	7	RPL27A Ribosomal protein L27a	IPI00587714	1	2
TACSTD1 Epithelial cell adhesion molecule	IPI00589818*	1	7	C12orf10 Chromosome 12 open reading frame 10	IPI00588179*	1	2
PSMD5 26S proteasome non-ATPase regulatory subunit 5	IPI00582424*	1	7	NUMA1 Nuclear mitotic apparatus protein 1	IPI00590550*	1	2
NANS Sialic acid synthase	IPI00573236*	1	6	VAT1 similar to Vesicle amine transport protein 1 homolog	IPI00591027*	1	2
DNAJB11 DnaJ (Hsp40) homolog, subfamily B	IPI00571322	1	6	CNOT1 similar to CCR4-NOT transcription complex, subunit 1	IPI00596498*	1	2
PPP1R7 Protein phosphatase 1 regulatory subunit 7	IPI00574127*	1	5	CARKD Carbohydrate kinase domain containing	IPI00596628*	1	2
LOC429867 Plectin-1	IPI00587768*	1	5	PAFAH1B1 Platelet-activating factor acetylhydrolase IB subunit alpha	IPI00596826*	1	2
WDR61 WD repeat-containing protein 61	IPI00584957*	1	5	SEC23B Protein transport protein Sec23A	IPI00601344	1	2
GNAI1 Guanine nucleotide-binding protein G(i) subunit alpha-1	IPI00585976*	1	5	RPL4 Ribosomal protein L4	IPI00575596	1	2
LOC396473 Myristoylated alanine-rich C-kinase substrate	IPI00591767	1	5	FARSA Phenylalanyl-tRNA synthetase alpha chain	IPI00584214	1	2
GOLPH3 similar to trans-Golgi protein GMx33	IPI00603208*	1	5	HDGF similar to hepatoma-derived growth factor	IPI00812721	1	2
RCJMB04_9n20 Isocitrate dehydrogenase [NAD] subunit beta	IPI00604247*	1	5	CENPT Centromere protein T	IPI00586160	1	2
PSMD2 26S Proteasome non-ATPase regulatory subunit 2	IPI00592623*	1	4	SPARC Osteonectin	IPI00575874	1	2

Listed are proteins that were exclusively identified in non-sensory cells as well as the number of times they were observed in three independent experiments and the sum of their spectral counts.

In AM1-43-low cells, we identified five proteins in all three experiments: T-complex protein 1 (CCT5), Annexin A6 (ANXA6), otolin-1 (OTOL1), protein phosphatase 2 (PPP2R1B) and delta-aminolevulinic acid dehydratase [24]. Otolin has previously been reported in supporting cells [25], an indication that the AM1-43-low cell population contained supporting cells.

Because AM1-43 is specifically taken up by hair cells, we were surprised that some previously described specific hair cell marker proteins, such as otoferlin, calretinin or parvalbumin were not only observed in the presumptive hair cell fraction, but were also detected in the AM1-43-low presumed non-sensory cell samples. A possible explanation for this potential contamination is that the mechanoelectrical transduction apparatus of some hair cells might have become damaged during the dissection, thereby leading to a fraction of unlabeled hair cells that would have been sorted into the AM1-43-low cell fraction.

To address this issue, we quantitatively assessed the expression profiles in the two different cell types with spectral counting and statistical testing of the contingencies of individual protein classifications into the two groups. We categorized the samples based on the assumption that a protein would be specific for either the hair cell or supporting cell population, which can be tested with Fisher’s exact analysis, generating a p-value for each identified protein. By far, the most abundant protein in the hair cell fraction was otoferlin (OTOF), a protein defective in a human deafness form DFNB9, and involved in the exocytosis and replenishment of synaptic vesicles to specialized ribbon synapses in hair cells [26]–[28]. Based on the significantly higher abundance of OTOF in the AM1-43-high cell population (321 spectral counts versus 17 spectral counts in the AM1-43-low population), we were confident that otoferlin is specific for the AM1-43-high cell population and that its expression in AM1-43-low cells is either caused by contaminating unlabeled hair cells or by low-level expression of otoferlin in non-sensory cells. Other proteins that were re-categorized to the hair cell population using Fisher’s exact test included adenylate kinase isozyme 1 (AK1), an enzyme involved in energy metabolism [29], the synaptic vesicle protein V-type proton ATPase subunit B (ATP6V1B2), as well as the hair cell markers calretinin [30], [31] and parvalbumin [32], [33]. Besides the lack of dye uptake by certain hair cells and the resulting potential contamination of the AM1-43-low population, we also reason that a single round of flow cytometry, even with discarding 20-25% of cells that were neither AM1-43 high nor low (Fig. 3C) results in >90% enrichment, but not absolute purity. Double sorting, on the other hand, would have dramatically reduced the cell number and thereby affected the overall detection sensitivity. Based on the results of the Fisher’s exact analysis, however, with a cutoff at a p-value of <0.05, we were able to reassign 53 additional proteins to the hair cell fraction (Table 3). Our interpretation of these assignments is that these 53 proteins are either hair cell specific or strongly upregulated in hair cells compared to non-sensory cells.

**Table 3:** Highly enriched hair cell proteins.

Up-regulated Hair Cell Proteins	Accession Number	Experiments Observed (total of 6)	Sum Spectral Count (HC)	Sum Spectral Count (NSC)	p-Value (Fisher Exact)
OTOF similar to brain otoferlin	IPI00599487	5	321	17	0
AK1 Adenylate kinase isoenzyme 1	IPI00571711	4	17	1	0.000023
ATP6V1B2 V-type proton ATPase subunit B, brain isoform	IPI00584789	3	14	1	0.00019
THOC4 THO complex 4	IPI00576073	4	13	1	0.00039
RPL10A 60S ribosomal protein L10a	IPI00596886	3	12	1	0.00078
CALB2 Calretinin	IPI00598353	5	107	9	7.9E-26
RPS10 Ribosomal protein S10	IPI00584482	3	11	1	0.0016
OCM2 Parvalbumin	IPI00602026	5	30	3	0.0000001
ATP6V1E1 ATPase, H+ transporting, lysosomal 31kDa, V1 subunit E1	IPI00583177	4	10	1	0.0031
FKBP3 FK506 binding protein 3	IPI00588963	4	10	1	0.0031
RAB14 Ras-related protein Rab-14	IPI00582881	3	9	1	0.006
ARL6IP5 ADP-ribosylation-like factor 6 interacting protein 5	IPI00597483	3	9	1	0.006
PEBP1 similar to Phosphatidylethanolamine-binding protein 1	IPI00603045	4	16	2	0.00024
SNAP91 Clathrin coat assembly protein AP180	IPI00595127	3	8	1	0.012
HSD17B10 Hydroxysteroid (17-beta) dehydrogenase 10	IPI00598537	4	8	1	0.012
ATP5F1 ATP synthase B chain	IPI00570686	4	22	3	0.00002
UCHL1 Ubiquitin carboxyl-terminal hydrolase isozyme L1	IPI00595105	3	7	1	0.023
DCI similar to Dodecenoyl-Coenzyme A delta isomerase	IPI00591896	4	7	1	0.023
MYO6 Isoform 1 of Myosin-VI	IPI00572880	5	150	22	4.9E-29
YWHAB 14-3-3 protein beta/alpha	IPI00591852	4	33	5	0.00000028
317 kDa protein Lipopolysaccharide-responsive and beige-like anchor protein isoform 2	IPI00580943	4	19	3	0.00013
ATP6V0A1 V-type proton ATPase	IPI00818110	4	6	1	0.043
ALDH2 Aldehyde dehydrogenase 2 family (mitochondrial)	IPI00589575	4	28	5	0.0000062
RCJMB04_15c3 Vesicle-trafficking protein SEC22b	IPI00583615	4	11	2	0.0058
MAP1B Microtubule-associated protein 1B	IPI00823023	4	41	8	0.000000089
ATP5O ATP synthase	IPI00813389	3	9	2	0.019
ME1 Malic enzyme	IPI00577117	3	9	2	0.019
HSPA4L Heat shock 70kDa protein 4-like	IPI00573597	3	9	2	0.019
RAB11B	IPI00573563	3	9	2	0.019
INPP5K Inositol polyphosphate 5-phosphatase K	IPI00601849	3	9	2	0.019
RAB1A	IPI00684373	5	26	6	0.000062
CBR1 20-hydroxysteroid dehydrogenase	IPI00577014	4	8	2	0.035
SOD1 Superoxide dismutase [Cu-Zn]	IPI00598533	3	8	2	0.035
RCJMB04_24f23 Endoplasmic reticulum resident protein 29	IPI00597655	5	11	3	0.016
ATP6V1A ATPase, H+ transporting, lysosomal 70kDa, V1 subunit A	IPI00579550	4	10	3	0.027
ARF1 ADP-ribosylation factor 1	IPI00822785	4	56	18	0.00000033
RCJMB04_1d23 Rho GDP dissociation inhibitor (GDI) alpha	IPI00585707	5	18	6	0.0045
CLTCL1 similar to Clathrin, heavy polypeptide	IPI00683666	4	112	41	9.3E-10
SLC25A3 Solute carrier family 25 member 3	IPI00573447	5	12	5	0.041
CLTC clathrin heavy chain 1	IPI00829409	6	295	124	2.6E-23
PHB Prohibitin	IPI00574627	4	20	9	0.013
ACLY ATP citrate lyase	IPI00575808	4	33	15	0.0016
SLC25A6 ADP/ATP translocase 3	IPI00600989	6	33	18	0.007
CKB Isoform Bb-CK-2 of Creatine kinase B-type	IPI00604016	6	236	131	5E-12
RCJMB04_11l21 14-3-3 protein zeta	IPI00578632	6	45	27	0.0048
GSTA3 Glutathione S-transferase	IPI00596765	5	25	15	0.032
TPI1 Triosephosphate isomerase	IPI00582452	6	41	26	0.012
LOC429558 similar to histone H2B	IPI00600992	6	51	34	0.009
PPIA Peptidyl-prolyl cis-trans isomerase	IPI00953851	6	37	26	0.036
YWHAQ 14-3-3 protein theta	IPI00577739	6	34	24	0.044
TUBB3 Tubulin beta-4 chain	IPI00603718	4	120	93	0.00058
DYNC1H1 similar to dynein, cytoplasmic, heavy polypeptide 1	IPI00575860	5	153	127	0.0041
TUBA1C Tubulin alpha-1 chain (Fragment)	IPI00575989	6	167	152	0.022

All proteins with a Fishers exact test p-value less than 0.05 are listed for proteins that are much more abundant in hair cells than in non-sensory cells, indicative of significant enrichment in hair cells.

Conversely, we also found proteins that are strongly upregulated or specifically detectable in the non-sensory cell fraction, based on the same assumptions and Fisher’s exact test with a cutoff of p<0.05, we reassigned 68 proteins to the non-sensory cell fraction (Table 4).

**Table 4:** Highly enriched non-sensory cell proteins.

Up-regulated Non-Sensory Cell Proteins	Accession Number	Experiments Observed (total of 6)	Sum Spectral Count (HC)	Sum Spectral Count (NSC)	p-Value (Fisher Exact)
SCCPDH Saccharopine dehydrogenase (putative)	IPI00580273	4	1	34	8.9E-09
RCJMB04_2a4 ATP-dependent RNA helicase DDX3X	IPI00579247	4	1	14	0.0011
TLN1 Talin-1	IPI00586709	5	3	39	0.000000029
SEC31A Protein transport protein Sec31A	IPI00571140	2	1	12	0.0035
SMC3 Structural maintenance of chromosomes protein 3	IPI00598955	3	1	12	0.0035
TXNDC4 similar to Thioredoxin domain containing 4	IPI00679931	2	1	11	0.0061
PARP1 Poly (ADP-ribose) polymerase 1	IPI00588387	3	1	11	0.0061
COL14A1 Collagen alpha-1(XIV) chain	IPI00601719	2	1	10	0.011
SERPINB6 Serpin B6	IPI00572003	4	1	10	0.011
DCPS Decapping enzyme, scavenger	IPI00583720	2	1	10	0.011
AKR1B10 Aldo-keto reductase family 1 member B10	IPI00591510	3	2	19	0.00034
AKR1A1 Alcohol dehydrogenase [NADP+]	IPI00820020	3	1	9	0.018
ACTR2 Actin-related protein 2	IPI00585509	3	1	9	0.018
HDLBP Vigilin	IPI00820163	2	3	26	0.000034
DST Dystonin	IPI00573263	5	2	16	0.0016
GSN Gelsolin	IPI00582056	4	2	16	0.0016
PGM1 Phosphoglucomutase 1	IPI00735086	3	1	8	0.031
AHCY similar to S-adenosylhomocysteine hydrolase	IPI00600960	4	1	8	0.031
PRPSAP2 Phosphoribosyl pyrophosphate synthase-associated protein 2	IPI00590654	3	1	8	0.031
KPNB1 Importin subunit beta-1	IPI00603965	4	1	8	0.031
RCJMB04_7e11 Isocitrate dehydrogenase 2	IPI00577774	5	4	29	0.000028
MYH9 Myosin-9	IPI00572165	6	9	65	2.4E-10
VCL Vinculin	IPI00589062	5	8	56	6.1E-09
ACTN4 Alpha-actinin-4	IPI00572461	6	4	25	0.0002
PGD 6-phosphogluconate dehydrogenase, decarboxylating	IPI00570964	3	2	11	0.02
PDHB similar to Pyruvate dehydrogenase (lipoamide) beta	IPI00601873	3	2	11	0.02
IDH1 similar to cytosolic NADP-dependent isocitrate dehydrogenase	IPI00598809	5	9	49	0.00000059
AKR1B1 Aldose reductase	IPI00591295	3	4	19	0.0035
NCL Nucleolin	IPI00680028	5	3	14	0.013
SEPHS1 Selenophosphate synthetase 1	IPI00576653	2	3	14	0.013
HSPD1 HSP60	IPI00577421	5	9	40	0.000035
HSP90AB1 Heat shock protein HSP 90-beta	IPI00820593	4	11	47	0.037
SERPINH1 Serpin H1	IPI00600018	4	13	53	0.0000047
EEF2 Elongation factor 2	IPI00585747	6	17	69	0.00000019
ANXA8L1 Annexin VIII	IPI00585409	4	4	16	0.013
HBG1;HBG2 Hemoglobin subunit beta	IPI00590350	4	3	11	0.05
CANX Calnexin	IPI00603318	5	3	11	0.05
Histone H1.03	IPI00571411	5	6	21	0.008
CTNNA1 Catenin alpha-1	IPI00600729	5	4	14	0.03
IQGAP1 RasGAP-like with IQ motif	IPI00571767	4	8	26	0.0048
PDIA6 Protein disulfide-isomerase A6 precursor	IPI00586516	5	10	31	0.0029
ALDH1A3 Retinaldehyde dehydrogenase 3	IPI00684362	5	6	18	0.025
GNB2L1 Guanine nucleotide-binding protein subunit beta-2-like 1	IPI00596315	5	5	15	0.041
ATP1A1 ATPase, Na+/K+ transporting, alpha 1 polypeptide	IPI00588683	5	26	71	0.000048
ANXA5 Annexin A5	IPI00592470	6	42	114	0.00000033
GOT1 Aspartate aminotransferase, cytoplasmic	IPI00589564	4	7	19	0.033
EIF4A2 Eukaryotic initiation factor 4A-II	IPI00588868	6	18	47	0.0014
PDIA4 Protein disulfide-isomerase A4 precursor	IPI00589958	4	17	44	0.0021
HSP90B1 Endoplasmin	IPI00570770	6	49	126	0.00000032
TCP1 T-complex protein 1 subunit alpha	IPI00584300	4	7	18	0.046
ACAT1 Acetyl-CoA acetyltransferase 1	IPI00579109	5	9	23	0.026
P4HB Protein disulfide-isomerase	IPI00596673	5	19	48	0.0017
IPO5 similar to Ran_GTP binding protein 5	IPI00572635	3	8	20	0.04
COPA Coatomer subunit alpha	IPI00577325	5	12	29	0.018
ACO2 Aconitase 2	IPI00576187	5	15	33	0.022
FLNB Filamin B	IPI00578831	5	78	170	0.00000073
MYH10 Nonmuscle myosin 10	IPI00576130	5	41	79	0.0037
FLNB Filamin	IPI00576318	3	66	127	0.00027
HSPA5 78 kDa glucose-regulated protein precursor	IPI00590375	6	76	140	0.00041
H4-VII Histone H4	IPI00572919	5	33	58	0.029
MDH2 similar to Malate dehydrogenase 2, NAD	IPI00577857	6	39	68	0.021
TPRXL Putative protein TPRXL	IPI00820086	4	87	147	0.0021
EEF1A1 Elongation factor 1-alpha 1	IPI00589985	6	67	108	0.016
VYGIII Vitellognin 3	IPI00818934	5	84	132	0.013
SERPINB14 Ovalbumin	IPI00583974	4	148	230	0.0019
GAPDH Glyceraldehyde-3-phosphate dehydrogenase	IPI00594653	6	123	188	0.0068
RCJMB04_1h13 Actin, cytoplasmic type 5	IPI00572084	6	225	309	0.02
TUBB2C Tubulin beta-3 chain	IPI00580626	5	155	167	0.00054

All proteins with a Fishers exact test p-value less than 0.05 are listed for proteins that are much more abundant in non-sensory cells than in hair cells, indicative of significant enrichment in non-sensory cells.

Our results revealed the limitation of our strategy that even minor cross-contaminations of the different cell types can mask the specific categorization of proteins. For proteins that are abundantly expressed in one cell type and not or only at a low level in the other, the spectral counting in combination with the Fisher’s exact test turned out to be a valuable tool to re-assign proteins to a single cell type.

### Categorization of the hair cells’ and non-sensory cells’ proteomes

**Figure 5:**
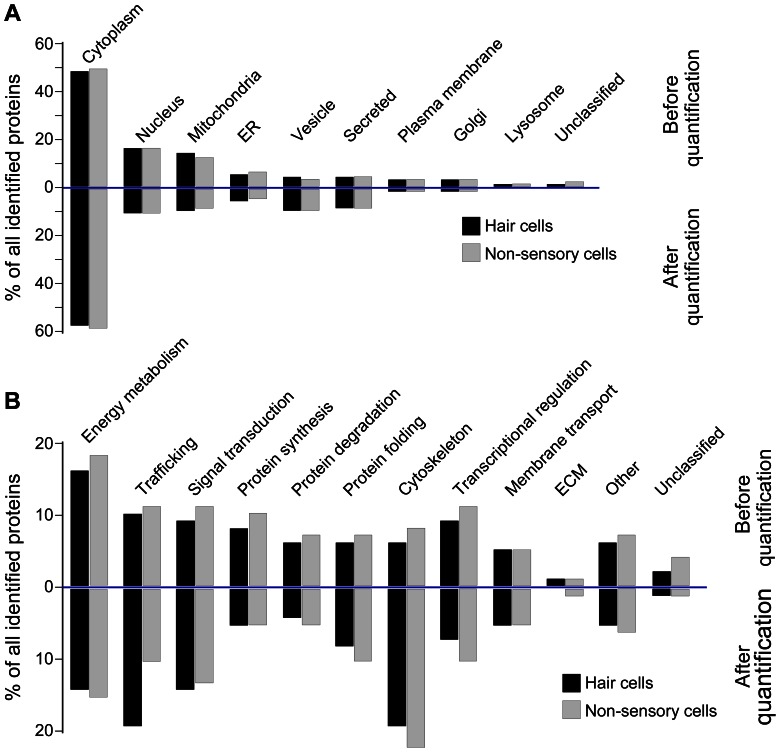
Categorization of subcellular localization and predicted cellular function of identified hair cell and non-sensory cell proteins. (A) All 634 identified hair cell and non-sensory cell proteins were classified according to gene ontology annotations and classified according to their subcellular localizations (upper bar graphs, before quantification). Taking into account the spectral counts of each identified protein resulted in slight changes of the distribution (lower bar graphs, after quantification). (B) Display of the gene ontology classifications of predicted cellular functions (upper bar graphs, before quantification) and after taking into account the spectral counts of each identified protein (lower bar graphs, after quantification).

To characterize the actual proteins that we identified with our proteomic approach in more detail, we subcategorized all identified proteins according to their annotated subcellular localization (Fig. 5A) and function (Fig. 5B). With respect to all proteins that we detected in hair cells and all proteins detected in non-sensory cells, we found no significant differences in proteome composition (Fig. 5A, before quantification). This was not very surprising because the majority of identified proteins (467) were observed in both cell types (see Fig. 4C). Nearly 50% of all identified proteins were cytoplasmic, 16% were nuclear and 13% were of mitochondrial origin. The residual 21% localized to vesicles, plasma membrane, Golgi apparatus, lysozymes or are secreted proteins. A small portion of proteins was not annotated and could not be assigned to a subcellular localization. Regarding function, the largest fraction (16–18%) of proteins identified in both cell types was found to be involved in energy metabolism, followed by trafficking, signal transduction, protein synthesis and degradation (Fig. 5B, before quantification). 1% of all identified proteins of each cell type function as extracellular matrix proteins.

Next, we conducted the same analysis but implied the spectral counts of each identified protein in order to compare relative expression levels. The subcellular distribution of all proteins was still comparable between hair cells and supporting cells (Fig. 5A, after quantification), however the ratios between subcellular compartments changed. Whereas an increase of 6% and 9% points was detected for the cytoplasmic localization, as well as vesicular and secreted proteins, respectively, a slight decrease was observed for the other subcellular compartments compared to before quantification.

Similar results were obtained for the analysis of the cellular functions. Here, a major increase was observed for cytoskeletal proteins that maintain the cellular structure for both hair cells and non-sensory cells (Fig. 5B, after quantification). A substantial difference between hair cell and non-sensory cell proteins appeared for the category trafficking. Whereas the percentage of non-sensory cell proteins involved in trafficking nearly stayed constant compared to before quantification, an increase of 9% points was noted for hair cells proteins. This result might be indicative of a potential higher need for protein trafficking in hair cells compared with non-sensory cells. Besides trafficking of stereociliary bundle proteins, hair cells also maintain substantial trafficking of proteins to the basolateral wall and synaptic sites. Interestingly, the most abundant protein we identified in the presumed hair cell fraction was otoferlin, which plays a key role in replenishment of synaptic vesicles in hair cells [26]. For non-sensory cells, an upregulation of extracellular matrix (ECM) proteins was noted after quantification (1% versus 0% points in hair cells), which could be an indication that these cells such as the supporting cells that are included in this population are closer associated with the basilar membrane compared to hair cells, or reflective of a possible contamination of this cell population with mesenchymal cells from the underlying stroma.

In summary, our analyses revealed differences in the proteomic compositions of chicken vestibular hair cells and non-sensory cells, which is not surprising given the specific function associated with sensory hair cells compared to non-sensory cell types. Our quantitative assessment of the data and the comparison is further limited by the fact that only the measureable portions of the proteomes are being considered, which creates a bias for abundant proteins detectable with the methods used.

**Figure 6:**
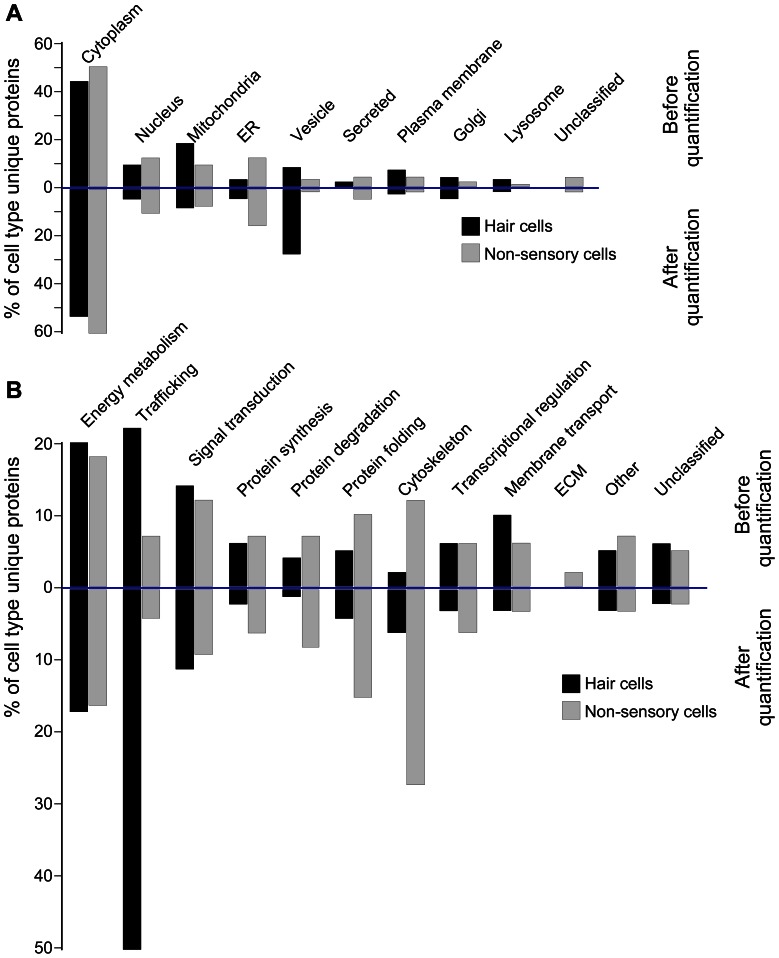
Categorization of subcellular localization and predicted cellular function for proteins unique to or highly enriched in hair cells and non-sensory cells. (A) Shown is the subcellular distribution of 116 proteins specific to or enriched in hair cells (Table 1) compared to 170 proteins exclusively identified or enriched in non-sensory cells (Table 2) (upper bar graphs, before quantification). Taking into account the spectral counts of each identified protein resulted in changes of the distribution (lower bar graphs, after quantification). (B) Display of the gene ontology classifications of predicted cellular functions of the proteins unique to hair cells and supporting cells (upper bar graphs, before quantification) and after taking into account the spectral counts of each identified protein (lower bar graphs, after quantification).

Based on these considerations, we hypothesized that potential differences between the two populations would be even more obvious if we focus our analysis on proteins that are either exclusively detectable in each group (Tables 1 and 2) and proteins that are highly enriched or specific for each group (Tables 3 and 4). Comparisons with respect to subcellular localization revealed that of the specific hair cell and non-sensory cell proteomes 40% to 50% of all unique proteins were of cytoplasmic origin (Fig. 6A, before quantification). A higher percentage of unique non-sensory cell proteins over unique hair cell proteins was assigned to the ER (12% compared to 3% for hair cell specific proteins), or were not annotated. In contrast, slightly higher percentages of unique or upregulated hair cell proteins were found to be of mitochondrial, vesicular, Golgi and lysosomal origin. After quantification, a main difference arose for the vesicle proteins where an increase of 26% points was revealed for unique or upregulated hair cell proteins compared to 1% of unique or upregulated non-sensory cell proteins (Fig. 6A, after quantification). This increase mainly arose from the high number of spectral counts of the two proteins otoferlin and clathrin, both shown to be involved in hair cell vesicle trafficking [26], [34]. Accordingly, for the cellular function, a notably strong upregulation was observed for hair cell specific proteins involved in trafficking to 50% of all hair cell specific/enriched proteins versus 4% of all non-sensory cell specific/enriched proteins (Fig. 6B, after quantification). These quantitative assessments demonstrate that in comparison to non-sensory cells, protein trafficking is strongly reflected in the hair cell proteome. As discussed earlier, this might reflect the high turnover of synaptic vesicles due to sustained exocytosis at the ribbon synapses, with otoferlin as a key player in vesicle recycling and replenishment, as well trafficking of proteins to the stereociliary hair bundle. Conversely, based on the quantification, the non-sensory cells’ proteome appears to be enriched for proteins involved in synthesis, degradation, folding and particularly cytoskeletal proteins, which could be an indication for a higher protein turnover and cytoskeletal specializations in these cells, despite the well-known cytoskeletal structures of hair cells.

### Validation of the proteomic analyses with immunohistochemistry

**Figure 7:**
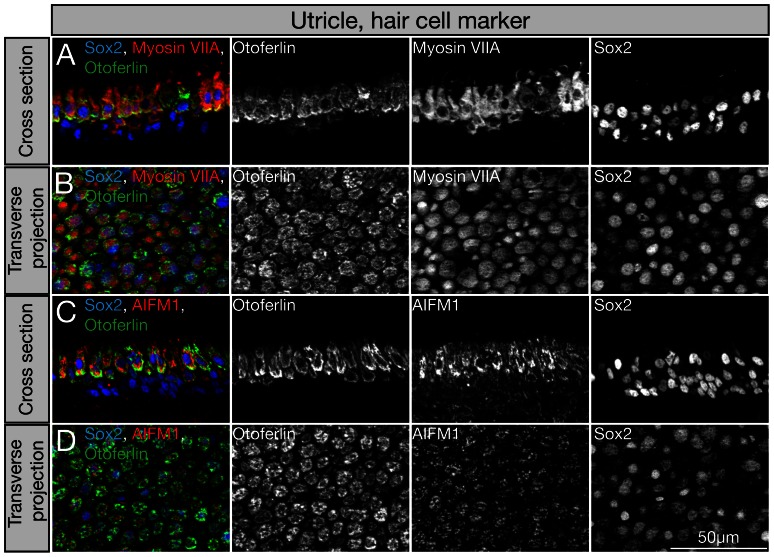
Qualitative analysis of identified hair cell markers by immunocytochemistry. Shown are cross sections of E18 chicken utricles (A and C) and transverse projections of utricle whole mounts (B and D). (A, B) Co-immunolabeling of the identified hair cell markers otoferlin (green), myosin VIIA (red), and Sox2 (blue), which is detectable in hair cells and supporting cells. (C, D) Co-immunolabeling with antibodies to otoferlin (green), the identified hair cell marker AIFM1 (red), and Sox2 (blue).

Not surprisingly, we identified a number of proteins in hair cells and non-sensory cells that previously were known markers for these cell types. Otoferlin for example, is a known hair cell protein [26], [35] that was identified in our analysis as highly enriched in hair cells after Fisher exact analysis. Monoclonal antibody staining confirmed that otoferlin is detectable in E18 by chicken utricular hair cells, co-labeled with antibodies to myosin VIIA, and that otoferlin immunolabeling is absent from non-sensory cells (Fig. 7A,B). We used Sox2 immunostaining to distinguish sensory epithelium cells from mesenchymal stromal cells. In the E18 chicken utricle, Sox2 protein is expressed by supporting cells and hair cells (Fig. 7A,B). We also confirmed hair cell expression of the mitochondrial protein apoptosis-inducing factor 1 (AIFM1), which was identified by our mass spectrometry analysis as hair cell only protein (Fig. 7C,D). The protein was, however, also detectable albeit with lower intensity in non-sensory cells. This result revealed, as previously discussed, a limitation of the comparative analyses that is a general lack of sensitivity for proteins that are not highly abundant. AIFM1, for example appears to be strongly enriched in hair cells and was identified via two independent peptides in one of the mass spectrometry experiments (Table 1). The protein was not detected by mass spectrometry in the non-hair cell fraction. Immunolabeling revealed a clear difference in staining intensity between hair cells and non-sensory cells, highlighting the differential expression of AIFM1 in these two cell types, but it also demonstrated expression of AIFM1 in non-sensory and supporting cells. This result shows that absence of detection of a protein in mass spectrometry does not mean that the protein is not present. Mass spectrometry has detection limits, which has been elegantly shown and discussed in a recent quantitative study of hair bundle proteins [21]. Overall, as reported in these recent results, we also observed that the detection limit for spectra is limited, leveling out at about 10^4^ per mass spectrometry run in the best cases. Particularly for abundant and large proteins, such a detection limit is not a big problem because the statistical likelihood that these proteins are represented by multiple peptides in a single run is quite high. For smaller proteins that are less abundant, the limit of detection might not be reached in a single run. In addition, it is reasonable to presume that simple biochemical features also limit the representation of certain groups of proteins – for example globular cytoplasmic *versus* membrane-spanning proteins, or detergent solubility, charge, protein degradation sensitivity, etc. For better representation and less variability, a substantial increase of the detection limit and methods for exclusion of abundant proteins would probably be the most efficient means.

**Figure 8:**
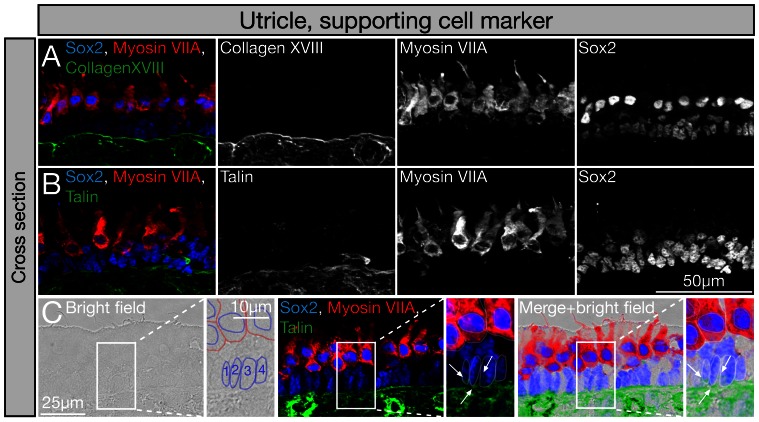
Qualitative analysis of identified non-sensory cell markers by immunocytochemistry. Shown are cross sections of E18 chicken utricles. (A) Co-immunolabeling with antibodies to collagen XVII (green), the hair cell marker myosin VIIA (red), and Sox2 (blue). Collagen XVII immunoreactivity was detected in the basal lamina and below the basal lamina in the mesenchymal stromal cell layer. (B) Antibodies to talin detected the protein in supporting cells, which are Sox2 (blue) immunopositive and myosin VIIA (green)-negative. Talin was also detectable in mesenchymal stromal cells. (C) To better visualize the location of talin immunoreactivity in supporting cells, we used light microscopic images and the Sox2 immunostaining to outline the supporting cells’ nuclei (labeled 1–4), and found association of talin immunoreactivity (green) perinuclear and toward the basal end of supporting cells (arrows).

For non-sensory cells, collagen XVIII alpha 1 and talin were identified by mass spectrometry, and monoclonal antibodies to these two chicken proteins detected them in association with non-sensory and supporting cells and not hair cells in the E18 utricle (Fig. 8). Antibodies to the extracellular matrix protein collagen XVIII [36], which was identified in the non-sensory cell only fraction (Table 2), labeled the basal lamina directly underneath the non-sensory cell layer. No immunoreactivity was detectable in hair cells. This localization, combined with the mass spectrometry data suggests that non-sensory and presumably supporting cells secrete collagen XVIII, but it cannot exclude a possible contribution of mesenchymal stromal cells to the mass spectrometry data because these cells were also labeled with collagen XVIII antibodies (Fig. 8A). The cytoskeletal protein talin, which is found concentrated at focal adhesion points and at points of cell-substratum contact [37], was identified via Fisher exact analysis as highly enriched by non-sensory cells compared to hair cells. Monoclonal antibodies detected the protein in supporting cells as well as in mesenchymal stromal cells, but not in hair cells (Fig. 8B,C).

### Concluding thoughts

We report a simple method to purify vestibular hair cells and non-sensory cells from the chicken inner ear. The approach generates cell populations of >90% purity that can be used for molecular studies, such as proteomic analyses. Our comprehensive evaluation of the individual datasets revealed certain limitations, such as presumptive inefficient labeling of damaged hair cells and the potential for cross-contamination during the single-pass flow cytometric sorting. On the other hand, we also showed that statistical analyses of proteomic data are a powerful tool to extract categorical information of protein distribution in experiments where minor cross-contamination affects the results. Our proteomic analyses identified proteins and protein categories that are enriched in vestibular hair cells and non-sensory cells. Some of these proteins were previously not considered in the context of inner ear sensory biology, and our datasets consequently are of relevance to researchers interested in hair cell and non-sensory or supporting cell function and development.

## Materials and Methods

### Dissociation of vestibular sensory epithelia into single hair cells and non-sensory cells

Embryonic day 18 (E18) chicken embryos were sacrificed by rapid decapitation. Utricles and saccules were dissected from the head in ice-cold HBSS with calcium and magnesium (Gibco) and otolithic membranes were removed without any enzymatic treatment. Next, utricles and saccules were carefully transferred with a micro spoon into AM1-43 dye solution (10 µM AM1-43 (Biotium) in Medium 199 (M199, Cellgro)) for 30 seconds at room temperature in a standard petri dish. Under these conditions AM1-43 preferentially enters hair cells via the mechanotransduction channel [13], [14]. After staining, utricles and saccules were transferred into fresh Medium M199 to wash off residual dye. A control sample of 5 utricles underwent the same staining procedure in AM1-43 dye free M199 medium.

Stained tissues were incubated in thermolysin (0.5 mg/ml; Sigma) in M199 for 30 minutes at 37°C and subsequently inactivated in M199 medium containing 5% FBS. The sensory epithelium was carefully dissected from the underlying stromal cells using a 1 ml 26-Gauge 1/2 needle syringe. We tested several dissociation conditions using various concentrations of trypsin, commercially available Accutase and Accumax (Innovative Cell Technologies), as well as an enzyme-free cell dissociation solution (Millipore, S-004-C). The most optimal conditions were determined by systematic pilot experiments. For enzymatic dissociation of sensory epithelia for flow cytometry, 120 sensory epithelia were incubated in 100 µl enzyme cocktail containing 50% Accumax and 0.025% trypsin/EDTA (Sigma) in M199 for 7 minutes at 37°C. After 3 minutes incubation, the tissue was gently disrupted by trituration for three times using a 1 ml syringe equipped with a 20-Gauge needle. After additional incubation for 4 minutes at 37°C, single cells were carefully dissociated by gently triturating the sample three to four times using a 26-Gauge needle attached to a 1 ml syringe. To stop trypsin activity, 0.25% trypsin inhibitor (Worthington) was added.

For subsequent fluorescent activated cell sorting, the cell suspension was passed through a 40 µm cell strainer (BD Falcon) to remove cell clumps.

### Immunocytochemistry

E18 chicken utricles and saccules were harvested in M199 (Gibco). For styryl dye uptake, the utricles were bathed in 10 µM FM1-43FX in M199, which is a fixable derivative of AM1-43 (Biotium) for 30 seconds and washed with M199 media (Invitrogen). The tissues were then transferred to 0.5 mg/ml thermolysin (Sigma) in M199 for 30 minutes at 37°C and then incubated in 5% serum for inactivation. Utricles and saccules were fixed with 4% paraformaldehyde in PBS for 30 minutes. The sensory epithelia were removed in 4% paraformaldehyde in PBS using a 1 ml 26-Gauge 1/2 needle syringe.

For immunocytochemistry, the tissues were fixed as described and cryoprotected overnight in 25% sucrose before embedding in OTC for cryosectioning. Serial sections (12 µm) were mounted on Ultrastick Gold Seal glass slides (Becton, Dickson) and dried completely. After an initial wash with PBS, the cryosections and whole mounts were incubated with 1% BSA (w/v) and 5% heat-inactivated goat serum in 0.1% Triton-100 PBS for 5 minutes. After blocking with 1% BSA in 0.2% Triton-100 PBS, cryosections and whole mounts were incubated overnight at 4°C with primary antibodies. Antibodies for detection of hair cell proteins were rabbit polyclonal myosin VIIA antibody (1∶1000; Proteus BioSciences, Inc.), guinea pig polyclonal antibody to myosin VIIA (1∶1000, [5]), mouse monoclonal antibody HCS-1 to otoferlin (1∶300 of hybridoma supernatant [35], [38], provided by Dr. Jeffrey T. Corwin (University of Virginia, Charlottesville, VA)), and a rabbit monoclonal antibody to AIFM1 (1∶200; Millipore, cat # 04-430). Supporting cells were immunolabeled with a goat polyclonal antibody to Sox2 (1∶200; Santa Cruz Biotechnology), a mouse monoclonal antibody to avian collagen XVIII (1∶10 of hybridoma supernatant of clone 6C4; DSHB), and mouse monoclonal antibody to avian talin (1∶10 of hybridoma supernatant of clone 8e6, DSHB). After washing with 0.2% Triton-100 in PBS, primary antibodies were detected with secondary antibodies: donkey anti-mouse Alexa647, donkey anti-rabbit Alexa488 and donkey anti-goat Alexa547 (Invitrogen) for 60 min at RT. For FM1-43FX labeled cryosections, we omitted detergents in all buffers used for immunocytochemistry. For each experiment presented, we analyzed at least 4 different embryos.

The tissues were mounted with ProLong® Gold Antifade Reagent (Life Technologies Corporation). Confocal images were acquired with a Zeiss Axioimager/LSM 5 Exciter confocal microscope. ApoTome images were captured on a Zeiss Axio Observer Z1 microscope with AxioVision software (Zeiss). Projection of z-stacks was performed with Axiovision software. Figures were prepared with Adobe Photoshop/Illustrator CS3.

### Fluorescence-activated cell sorting

Cells were analyzed using a BD Aria FACS cytometer. Control cells without AM1-43 staining that underwent the same conditions were first analyzed for background fluorescence activity. AM1-43 stained cells were then analyzed. Our gating strategy was as follows: 1) Exclude cells that take up the DNA intercalating dye propidium iodide, 2) Remove debris based on side scatter (SSC) and forward scatter (FSC) parameters, 3) Discard doublets based on FSC-height and FSC-area, and 4) Gate AM1-43-high cells (35.0±0.027%) and AM1-43-low cells (42.0±0.034%) for collection (see Fig. 3). A 100 µm flow cytometer nozzle size was used for all sorts in an effort to reduce cell damage. AM1-43-high and AM1-43-low populations were collected in lysis buffer (125 mM Tris-HCl, 50% glycerol, 4% SDS) in preparation for further analysis.

### Mass spectrometry

After separation of the AM1-43-high and AM1-43-low populations by FACS sorting, proteins were enriched by trichloroacetic acid (TCA, Sigma) precipitation. Briefly, one volume of ice-cold 50% trichloroacetic acid was mixed with one volume of cell suspension after FACS sorting and incubated over night on ice. Precipitated protein was collected by centrifugation at 38,000 x g (Sorvall SS34) for 30 minutes at 4°C. The supernatant was aspirated and the remaining pellet was washed with two volume of ice cold 90% acetone while incubating at –20°C for 45 minutes. After a second centrifugation at 38,000 x g (Sorvall SS34) for 30 min at 4°C, the supernatant was again aspirated and the pellet was air-dried. To dissolve the pellet 30 µl 8 M urea containing 100 mM DTT was added and incubated at 95°C for 5 minutes. After addition of 30 µl 2x Laemmli Buffer (62.5 mM Tris-HCl, 25% glycerol, 2% SDS, 0.01% Bromphenol Blue, pH 6.8), to the TCA precipitated protein sample, followed by 10 minutes incubation at 95°C, the samples were immediately applied completely on one gel lane of a 4–20% Bis/Tris Gradient Gel (Invitrogen). The gel was coomassie stained and each gel lane fractionated into 8 gel bands. The gel bands were in-gel digested using trypsin (Promega) and protease max (Promega) as previously described [39]. Three independent experiments were conducted resulting in 6 gel lanes analyzed. The extracted peptides were dried to completion using a speed vac, after which they were reconstituted using a buffer of 0.2% formic acid, 2% acetonitrile, 97.8% water. The HPLC was an Eksigent nano2D (Eksigent), in which a self-packed 150 µM ID C18 column was used. The electrospray source was either a Michrom Advance operated at 600 nL/min or an Advion Nanomate which was operated at 450 nL/min. Two mass spectrometers were used, an LCQ Deca XP+ and a LTQ Orbitrap Velos (Thermo Fisher). Data acquisition was done in a data dependent fashion in which the top 3 (Deca) or the top 10 (Velos) most intense multiply charged peptide ions were selected for MSMS fragmentation. In total three independent data sets were generated (N = 3) on each cell type, therefore 48 LC-MS/MS runs were interrogated.

The data were extracted using a msconvert script to mzXML format after which was searched using a Sorcerer (SAGE-N) search station employing the Sequest algorithm. Both, the NCBI *Gallus gallus* as well as the ipi chicken databases were searched. The LCQ Deca XP+ data was searched with a 1.2 Da mass window and the LTQ Orbitrap Velos data were searched using a 50 ppm mass window. We allowed for the static modification of propionamide on Cysteine and variable modifications of Methionine oxidation, Lysine acetylation, Serine, Threonine and Tyrosine phosphorylation as well as Lysine ubiquitination.
